# Gene expression profiling of progenitor cells isolated from rat rotator cuff musculotendinous junction

**DOI:** 10.1186/s12891-020-03190-9

**Published:** 2020-03-28

**Authors:** Mandeep S. Virk, Wei Luo, Katie J. Sikes, Jun Li, Anna Plaas, Brian J. Cole

**Affiliations:** 1 Department of Orthopaedic Surgery, Division of Shoulder & Elbow, New York University Langone Orthopedic Hospital, 301 East 17th street, New York, 10003 NY USA; 2 Dept of Internal Medicine, Rush University Medical Center, 1735 W Harrison St Jelke 1302, Chicago, 60612 IL USA; 3 Department of Clinical Sciences, Colorado State University, Translational Medicine Institute, Fort Collins, 80523 CO USA; 4 Department of Orthopaedic Surgery, Sports and Shoulder and Elbow Division, Midwest Orthopaedics at Rush University, 1611 West Harrison Suite 300, Chicago, IL USA

**Keywords:** Progenitor cell, Rotator cuff musculotendinous junction, Bone marrow cells, Rotator cuff tendon, Rotator cuff muscle, MSC gene expression profile

## Abstract

**Background:**

Rotator cuff tendon tears are typically degenerative and usually affect the region of tendon insertion on bone. The remnant torn tendon is degenerative and may not be an ideal source for progenitor cells for cell-based therapies. Therefore, the aim of this study was to determine if musculotendinous junction (MTJ), which is adjacent to tendon would be a viable alternate source of progenitor stem cells. We also sought to study the gene expression profile MTJ progenitors and compare it with progenitors isolated from RC tendon, RC muscle and other existing tissue sources (bone marrow, adipose tissue, and Achilles tendon).

**Methods:**

Rotator cuff tendon (RCT), muscle (RCM), and RCMTJ as well as Achilles tendon (AT) tissues were harvested from healthy male Lewis rats and progenitor cultures were established from these tissues and also from bone marrow and adipose tissue. Quantitative RT-PCR was performed on RNA extracts from intact tissues and progenitor cells using a custom array for the mesenchymal stem cell (MSC) differentiation marker genes. The gene expression profile of MSC differentiation markers within four tissues types, six progenitor cells, and between tissue and their corresponding progenitors were compared.

**Results:**

Progenitors cells can be isolated from rat rotator cuff musculotendinous tissue and their pattern of MSC gene expression was similar to the rotator cuff tendon progenitors for majority of the genes tested. However, there were significant differences between the MSC gene expression patterns of RCMTJ and RCM progenitors. Furthermore, there were differences in gene expression between the RCMTJ tissue and its progenitor cells with respect to MSC differentiation markers. The gene expression pattern of RCMTJ tissue was similar to RCM tissue with respect to markers of chondrogenesis, myogenesis, tenogenesis, and MSC specific markers.

**Conclusion:**

We demonstrate that the musculotendinous junction contains distinct set of progenitor cells and their MSC gene expression pattern is similar to rotator cuff tendon progenitors. RCMTJ progenitors will be an attractive option for cell-based regenerative treatment of chronic rotator cuff tears.

## Background

Rotator cuff (RC) tears are one of the most common causes of chronic shoulder pain, with approximately 250, 000 rotator cuff repairs performed in the United States annually [21]. While significant progress has been made in mechanical fixation of rotator cuff tears, failed rotator cuff repairs continue to be a challenge, especially with large tears [16]. Failed repairs of large and massive RC tears result in persistent shoulder pain and loss of shoulder function and eventually lead to shoulder arthritis and poor functional use of the arm [34, 39].

There is continued interest in biologic therapies to improve tendon to bone healing in rotator cuff repairs [2, 6, 29, 31]. The cell-based therapy using progenitor cells is a promising strategy for regeneration and healing of the tendon tissues [1, 6, 10, 11, 19, 23, 24, 26–28, 30, 36, 37]. However, the ideal progenitor cell type, or the critical growth factor involved in tendon-bone repair and ideal scaffold are not known. The tendon-derived progenitor cells (TPCs) have been previously isolated from patellar tendon, and rotator cuff tendon [3, 22, 28, 36, 37]. Rotator cuff tears are commonly degenerative and occur in the region of the tendon insertion. The remnant torn tendon is degenerative and may not be an ideal source for progenitor cells for cell-based therapies. Consequently, rotator cuff tendon is less attractive as a source of tendon progenitor cells for cell-based treatment of chronic rotator cuff tears. However, the adjacent musculotendinous junction is relatively preserved in chronic RC tendon tears and may be a viable alternate source of progenitor cells. The rotator cuff musculotendinous junction (RCMTJ) is an anatomic transition zone between a muscle and tendon and is a more vascularized region, which is less prone to wear and tear compared to tendon insertion in degenerative rotator cuff tears [13]. If we can isolate tendon progenitors from MTJ, it will be a viable option for harvesting tendon progenitors for cell-based therapies in patients with chronic rotator cuff tears.

In this study, we want to determine if there is a progenitor cell population in RCMTJ and also establish their gene expression profile for mesenchymal stem cell (MSC) differentiation markers. Our hypothesis is that progenitors cells do exist in rotator cuff musculotendinous junction and they are distinct population of cells different from the rotator cuff muscle tissue. Therefore, the purpose of this study was three folds; a) to isolate and characterize the progenitor cells from the rat RCMTJ b) to establish gene expression profile for MSC differentiation markers for native RMTJ tissue and compare it to the RCMTJ progenitors, and other tissues including rotator cuff tendon (RCT), rotator cuff muscle (RCM) and Achilles tendon (AT) and c) to compare the gene expression profiles of progenitor cells derived from RCMTJ, RCM, RCT and other tissues (bone marrow, adipose and Achilles tendon derived progenitor cells).

## Methods

This is a controlled laboratory study, which was performed using rat rotator cuff, Achilles tendon, bone marrow, and adipose tissue. All experiments were performed in triplicate.

### Rationale for experiments

Our first aim was to assess the feasibility of isolating progenitor cells from the rotator cuff musculotendinous junction tissue. Towards this we harvested tissue from the rat RCMTJ and isolated cells from the MTJ tissue. We used the basic property of plastic adherence of progenitor cells to selectively identify and expand the MTJ derived progenitor cell population and subsequently studied their morphology and MSC gene expression pattern.

Our second aim was to compare the MSC gene expression of native tissues (rotator cuff muscle, rotator cuff tendon and rotator cuff musculotendinous junction and Achilles tendon) with the progenitors derived from each corresponding tissue. Towards this, we compared the MSC gene expression profiles of intact tissue RNA extracts with the corresponding progenitors to characterize the differentiated tissue-specific cell population, that exist together with specific progenitors in each individual tissue. We also compared the gene expression of tissue extracts from the rotator cuff muscle, tendon and musculotendinous junction. Our hypothesis for this part of the experiment was that tissues in three locations (rotator cuff muscle, tendon, and musculotendinous junction) should have different MSC gene expression profiles if they are anatomically or functionally distinct zones.

Our third aim was to compare the MSC gene expression profiles of MTJ derived progenitors with the progenitors derived from the RC muscle and RC tendon to determine if there is a difference in gene expression at three different locations within the same tissue (rotator cuff). Additionally, we also compared the MSC gene expression pattern of RC-MTJ derived progenitors with progenitors derived from other tendon (Achilles tendon) and non-tendon sources (bone marrow and adipose tissue), which served as controls for these experiments.

### Ethics statement and animal euthanasia

We obtained ethical approval from the Institutional Animal Care and Use Committee of Rush University Medical Center.

### Tissue harvest

**Fig. 1:**
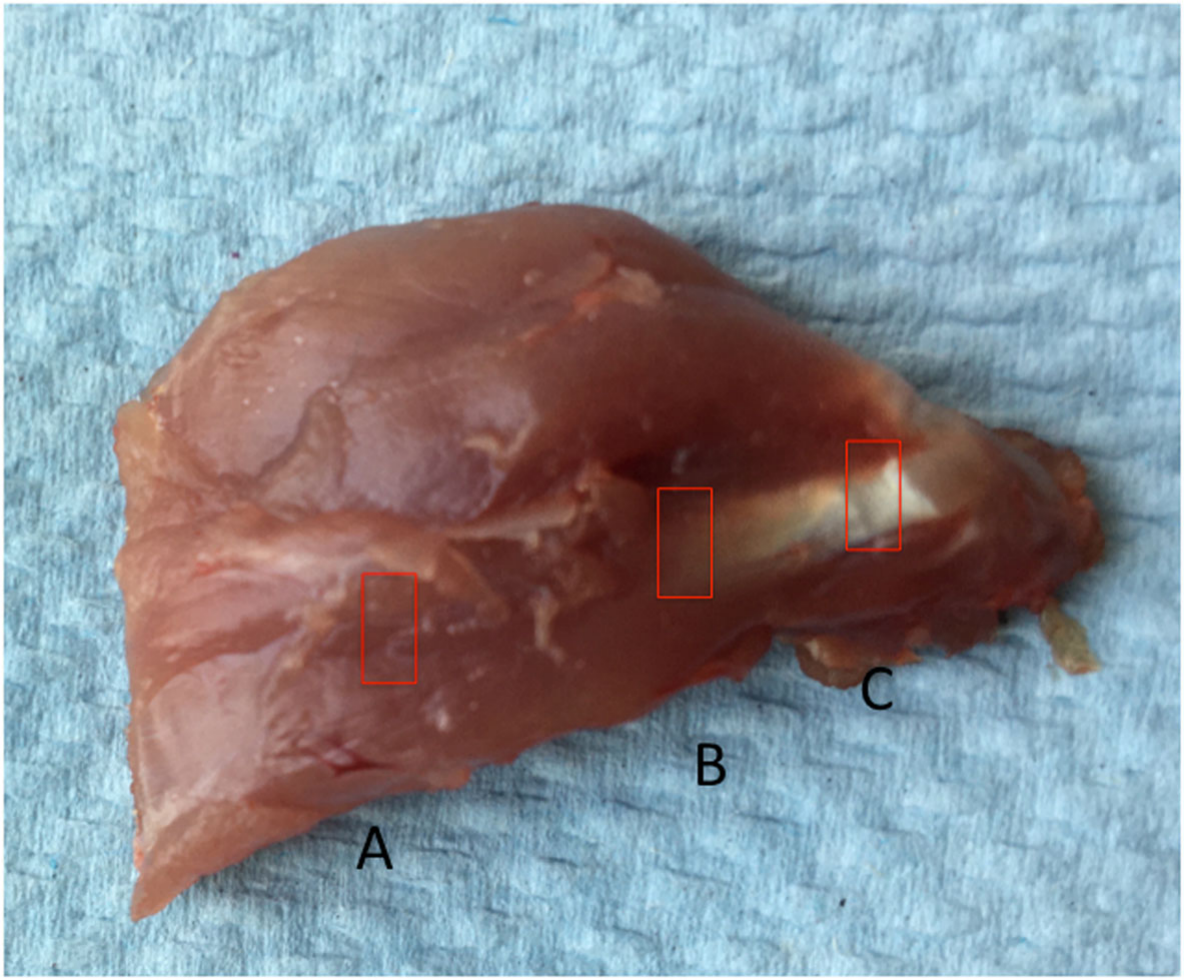
Rat rotator cuff tissue showing three different sites of tissue harvest for progenitor cell isolation [rotator cuff muscle (**a**), musculotendinous junction (**b**) and tendon insertion (**c**)]

A total of 9 male Lewis rats were used for this experiment (three rats per experiment and total of three experiments). The three experiments were performed on three different days. Tissue collections for RNA purification and cell isolation were performed immediately after sacrifice. The rotator cuff muscle, tendon (close to insertion), and musculotendinous junction tissues as well as the Achilles tendon were harvested from male mature Lewis rats (16–20 weeks old). The rotator cuff tissue was harvested after removing the skin, subcutaneous tissue, and deltoid from both shoulder girdles [8]. The overlying coracoacromial arch was removed to facilitate further exposure of rotator cuff tendons. The musculotendinous junction is a transition zone that contains a mix of muscle and tendon and spreads/extends over a broader area and not a single demarcation line. Notably, in the rat, rotator cuff muscle and tendon are sufficiently large to facilitate the macroscopic identification of this region. The RCT, RCM, and RCMTJ were delineated by visual inspection and tissue harvested using a surgical knife blade (Fig. 1). The harvested RCT, RCM, and RCMTJ tissue was immediately transferred to liquid nitrogen.

The Achilles tendon was harvested from both lower extremities as described previously [7]. A midline posterior incision was made on the hind limb of the animal. After subcutaneous dissection, the Achilles tendon was exposed and freed from the plantaris tendon and overlying paratenon. The Achilles tendon was cut from its insertion and harvested with a surgical knife blade avoiding any contamination from the musculotendinous junction. The harvested Achilles tendon tissue was immediately transferred to liquid nitrogen.

### Isolation and culture of murine progenitor cells

A total of 15 Lewis rats were used for this experiment (five rats per experiment and total of three experiments). Progenitor cells were isolated from Achilles tendon, bone marrow, adipose tissue, rotator cuff muscle, tendon, and musculotendinous junction. The adipose tissue (AdT) was harvested from the inguinal fat as described previously [25]. The RCM, RCT, RCMTJ, and AT tissues were harvested as described above. The harvested tissues (RCM, RCT, RCMTJ, AdT, and AT) were handled in a similar fashion for progenitor cell harvest and culture. The harvested tissue was minced and placed into CO2-independent medium (Gibco, Gaithersburg, MD) with 3 mg/ml collagenase type I (Worthington, Lakewood, NJ) and dispersed under agitation for 2 h at 37 °C. The digest was then centrifuged at 300 g for 10 min. The supernatant lipid-rich layer was discarded, and the pellet treated with 1.5 mL RBC lysis buffer (eBioscience, Waltham, MA). The pellet was then washed three times in PBS, before suspension in 60 mL of DMEM/5 mM glucose/10% FBS (Atlanta Biologics) containing 2 ng/mL bFGF, (R&D) and plated into two T75 flasks (Corning). The typical cell yield of the collagenase isolates was approximately 1–2^6 cells. However, we observed variable attachment of cells after the 24 h adherence phase. Thus, based on the density of adherent cells this varied between 0.5–1 × 10^6 cells per T75 flask. Nonadherent cells were removed after 24 h and fresh medium was added. Medium changes were performed every 48–72 h until cells reach confluency of ~ 90% between days 8–10 post plating. The cell yield after trypsinization of each T75 flask was approximately 6–6.5 × 10^6 cells. These cell suspensions were equally divided between three T25 flasks (Corning) and plated at the density of approximately 2–2.5^6 cells, which resulted in ~ 90% confluency immediately after cells adhered, thus minimizing additional proliferation of the passaged cells prior to RNA extractions.

Bone marrow was harvested from the femurs and tibias under sterile conditions according to a previously published protocol [38]. Briefly, the whole bone marrow was obtained under sterile conditions by flushing the marrow cavity with Iscove’s modified Dulbecco’s medium (Irvine Scientific, Santana, CA) in a 30 cc syringe and 18-gauge needle. The resulting cell suspension was centrifuged at 300 g for 10 min. The pellet was then suspended in 30 mL of medium (DMEM/5 mM glucose/10% FBS containing 2 ng/mL bFGF) and plated in T75 flasks and treated further like rotator and Achilles tendon cells.

General morphological characteristics during the primary culture and the subsequent passage were evaluated under light microscope. Specifically, elongated cell shapes, multiple plasma membrane extension, absence of apoptotic nuclear changes, intercellular spaces, and absence of dense packing and fibroblastic alignments were similar for both, primary and first passage cells.

### Quantitative real time PCR

RNA was isolated from the intact tissue (following fragmentation in a Bessman tissue pulverizer at − 196 °C) and from MSCs [passage 2 (P2)] via TRIzol® extraction (Invitrogen, Grand Island, NY) as previously described [4, 20, 33]. Briefly, RNA was purified with an RNeasy MiniKit (Qiagen, Valencia, CA) with RNA concentration and purity (A260/280 > 1.9) analyzed using a Nanodrop (ND-1000, Thermoscientific Waltham, MA). cDNA was synthesized using Qiagen RT2 First Strand kit. Quantitative RT-PCR gene expression of rat rotator cuff (RCM, RCT, and RCMTJ) and Achilles tendon (AT) tissue, and their corresponding cultured progenitors cells (RCMC, RCTC, RCMTJC, ATC) and other cultured progenitor cells (BMSC and ADSCs) were profiled using the Rat Mesenchymal Stem Cell RT^2^ Profiler™ PCR Array (PARN-082ZA, Qiagen, Valencia CA). A total of 84 genes were tested and included genes representing markers of stemness, chondrogenesis, tenogenesis, adipogenesis, osteogenesis, and MSC specific and other MSC markers.

### Data analysis

The data was expressed as fold change of mRNA abundance in RCT MTJ tissue and progenitor cells compared to other tissue or progenitor cells, which was calculated as follows. First, the ΔCt relative to the housekeeping gene B2m was calculated for each tissue and progenitor cell for each MSC gene. Then, the ΔΔCt which = ΔCt RCMTJ – ΔCt (reference tissue) and finally the fold change (2^-ΔΔCt) was calculated. For the tissue to cell comparison, the ΔΔCt ends up being ΔCt Cell – ΔCt tissue. All statistical testing was performed on the ΔCt values using Graph Pad Prism 5 (La Jolla, CA), and the fold changes are presented for easier interpretation. A 1-way ANOVA with Tukey’s post-hoc tests was utilized over other types of statistical testing as the primary focus for biological relevance 1) differences between different cell and tissue types, and 2) tissue and cell specific comparisons. A *p* value of *p* < 0.05 was considered significant.

## Results

### MTJ progenitor cell isolation, and growth and morphology (RCMTJC) in culture conditions

**Fig. 2:**
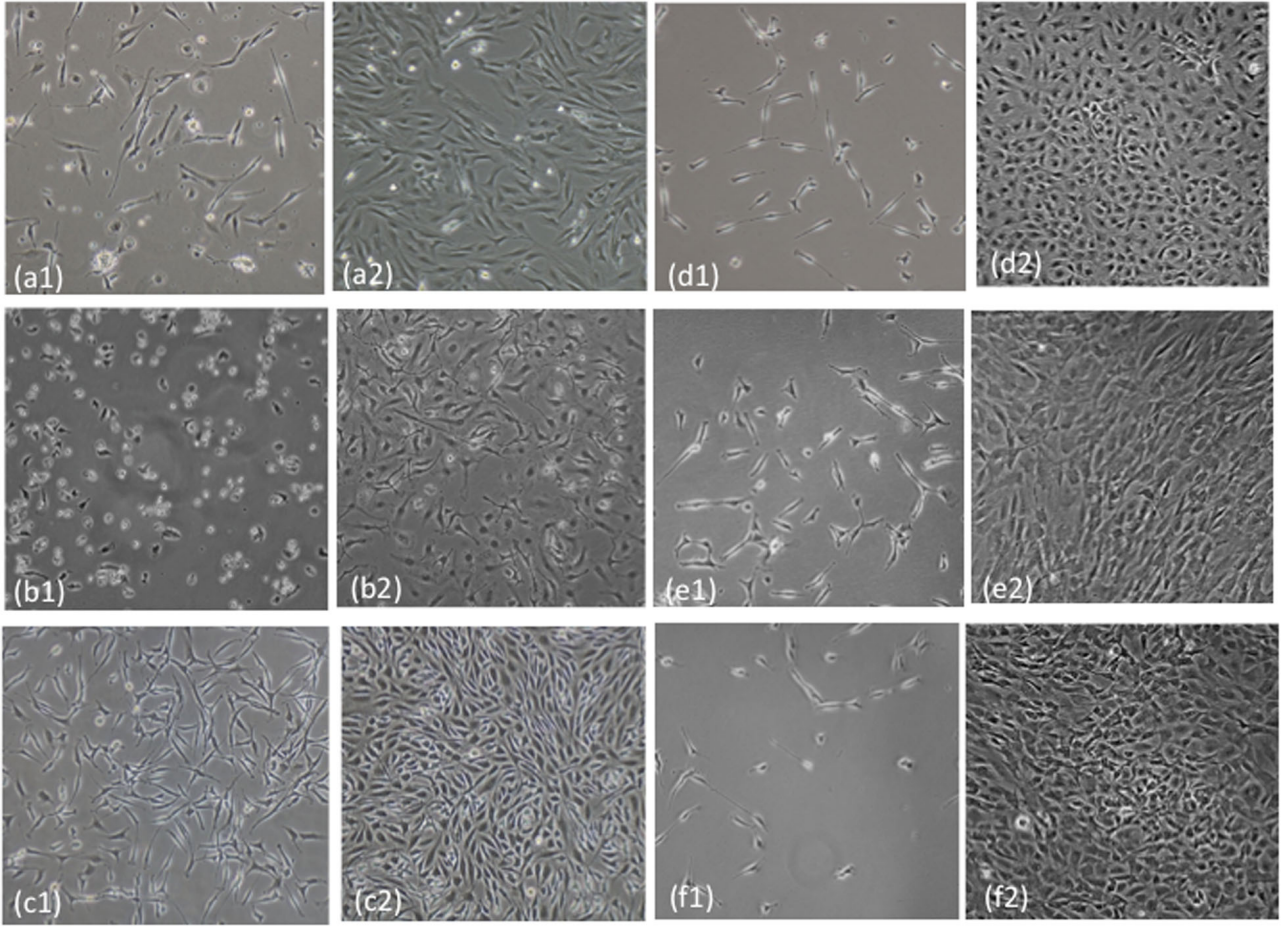
Phase contrast images of cultured stromal fibroblasts at 24 h post plating (a1-f1) and at confluency (a2-f2). Gene expression analyses were performed on confluent cells only. (a-ADSC; b-BMSC; c-AT; d-RCT; e-RCM; f-RCMTJ)

The progenitor cells from the rotator cuff MTJ (RCMTJC) were isolated using standard harvest and culture protocol similar to that used for isolating progenitors from rotator cuff muscle and tendon. We used the basic property of plastic adherence of progenitor cells to selectively identify and expand the MTJ derived progenitor cell population in tissue culture. There were no specific morphological characteristics of progenitors isolated from RCMTJCs and there were no significant differences in morphology and growth characteristics compared to progenitors isolated from RCM or RCT (Fig. 2).

### MSC gene expression profile of rotator cuff musculotendinous junction tissue

**Fig. 3:**
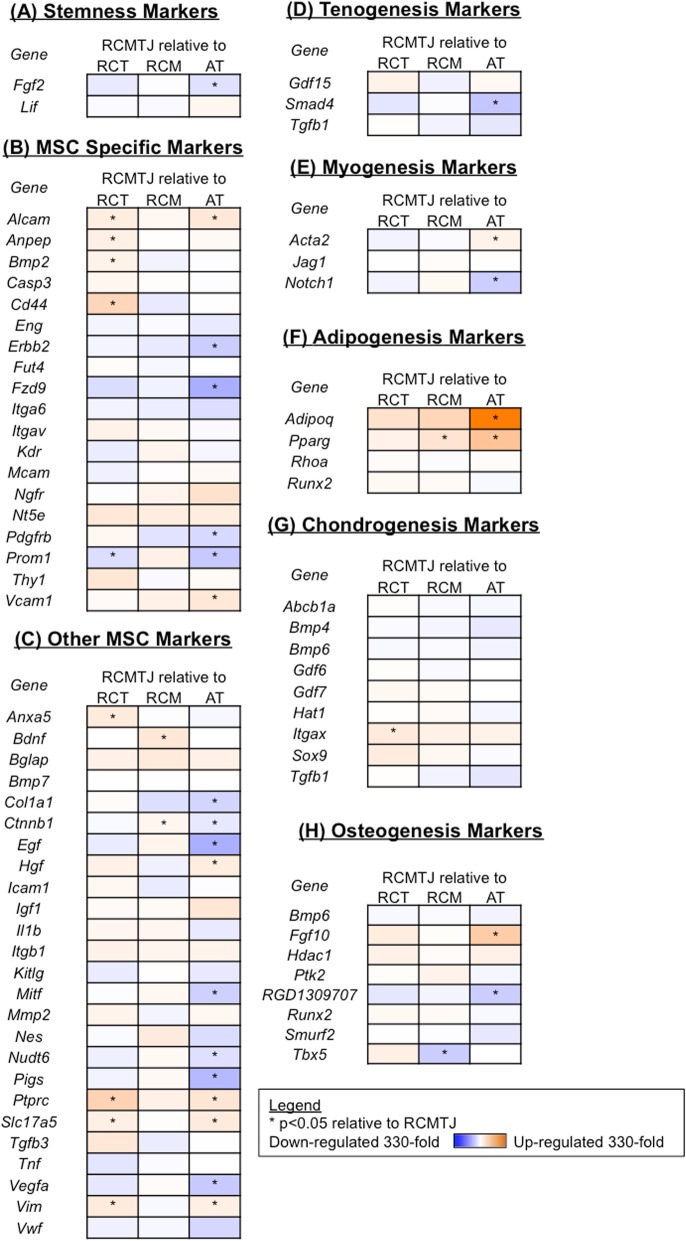
Heat map representation of the fold change in expression of (**a**) stemness markers, **b** MSC specific markers, **c** other MSC markers, **d** tenogenesis markers, **e** myogenesis markers, **f** adipogenesis markers, **g** chondrogenesis markers, and (**h**) osteogenesis markers for the comparison of rotator cuff myotendinous junction (RCMTJ) tissue to rotator cuff tendon (RCT) tissue, rotator cuff muscle (RCM) tissue, and Achilles tendon (AT) tissue. The following genes were not detected: Csf2, Csf3, Fut1, Gnf4, Hnf1a, Ifng, Il6, Il10, Ins2, Pou5s1, Sox2, and Tert

MSC gene expression profile of RCMTJ tissue was very similar to that of RCM tissue except for a significant increase in expression of one osteogenesis marker, *Tbx5,* and a significant decrease in expression of two MSC markers *(Ctnnb1 and Bdnf*) and one marker of adipogenesis (*Pparg*; Fig.3). Conversely, significant differences in gene expression of various MSC genes between the RCMTJ and RCT or AT were found (Fig.3). RCMTJ was similar to RCT for MSC differentiation markers for osteogenesis, myogenesis and tenogenesis but there was significant down regulation of MSC specific markers (*Alcam, Anpep, CD44,* and *BMP-2*), other MSC markers (*Anxa5, Ptprc, Slc17a5,* and *Vim*), and *Itgax*, a chondrogenesis marker. One of the MSC specific markers, *Prom1,* was upregulated in RCMTJ compared to RCT*.*

Compared to AT, the RCMTJ tissue had significant up regulation of number of genes including myogenesis marker, *Notch1*, tenogenesis marker, *Smad4,* osteogenesis marker, *RGD130970*, stemness marker, *Fgf2,* MSC specific markers, *Erbb2, Fzd9, Pdgfrb,* and *Prom1,* and other MSC markers *(Col1a1, Ctnnb1, Egf, Pigs, Nudt 6,Mitf, Vwf,* and *Vegfa).* However, AT tissue had significantly higher expression of MSC specific markers, *Alcam, Hgf, Vcam1, Hgf, Ptprc, Slc17a5* and *Vim,* and markers of adipogenesis (*Adipoq and Pparg),* and osteogenesis (*Fgf10).* There were no significant differences between the RCMTJ and AT with respect to markers of chondrogenesis.

### Differences between progenitor cell and tissue MSC gene expression in rotator cuff musculotendinous junction tissue

**Fig. 4:**
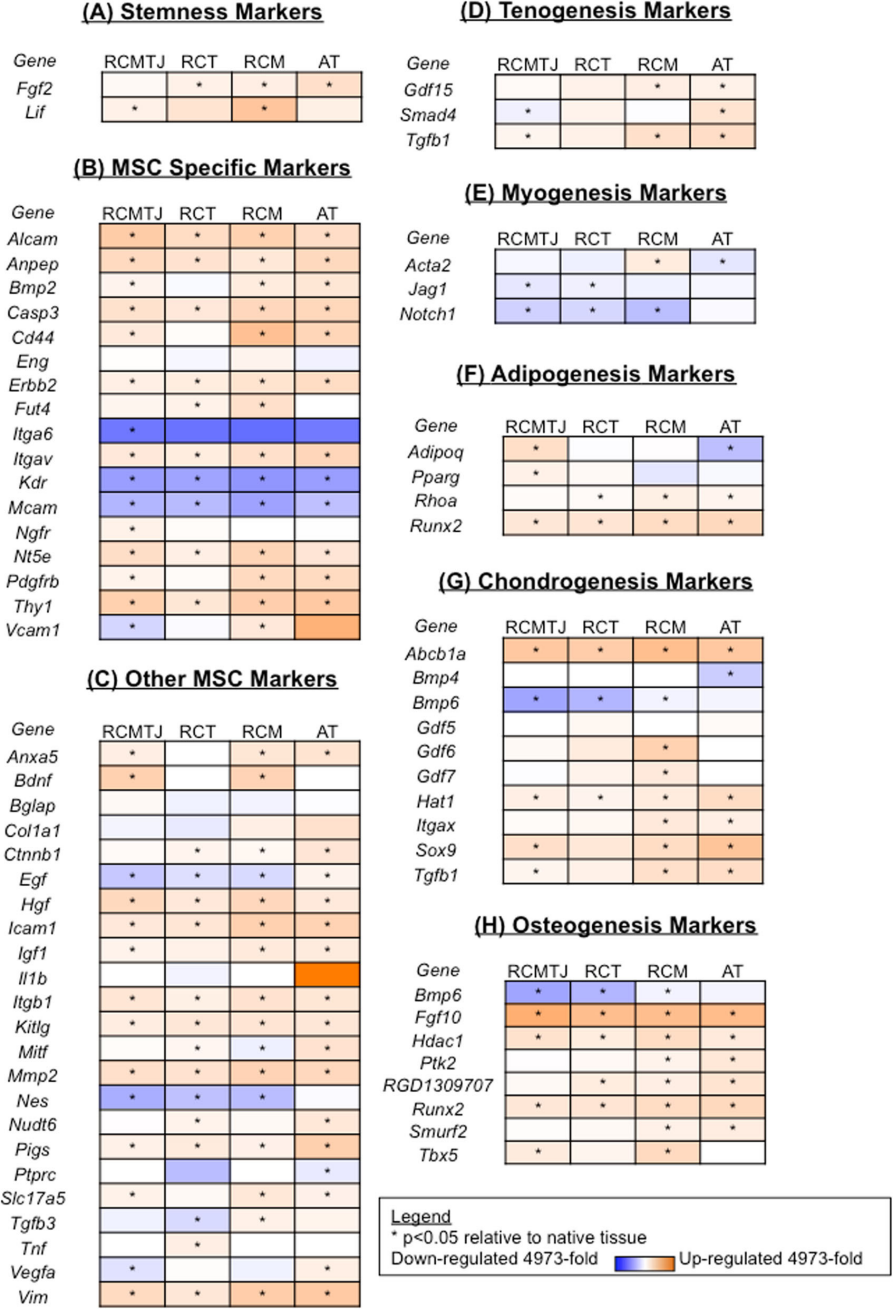
Heat map representation of the fold change in expression of (**a**) stemness markers, **b** MSC specific markers, **c** other MSC markers, **d** tenogenesis markers, **e** myogenesis markers, **f** adipogenesis markers, **g** chondrogenesis markers, and (**h**) osteogenesis markers for the comparison of progenitor cells relative to correponding tissue for rotator cuff myotendinous junction (RCMTJ), rotator cuff tendon (RCT), rotator cuff muscle (RCM), and Achilles tendon (AT). The following genes were not detected: Bmp7, Csf2, Csf3, Fut1, Fzd9, Hnf1a, Ifng, Il10, IL6, Ins2, Prom1, Pou5s1, Sox2, Tert, and Vwf

We compared the MSC gene expression profiles of intact tissue RNA extracts with the corresponding progenitors to characterize the differentiated tissue-specific cell population, that exist together with specific progenitors in each individual tissue (Fig. 4) Of the 84 genes tested, significant differences in expression of 45 genes (increased expression in 34 genes and decreased expression in 11 genes) were detected between the RCMTJ and its progenitor cell population (RCMTJC) as shown in Fig. 4. However, these differences between tissue and cells were not specific to RCMTJ and were also seen with other tissues (AT, RCT and RCM) and their corresponding progenitors.

### MSC gene expression profile of MTJ progenitor cells (RCMTJC)

**Fig. 5:**
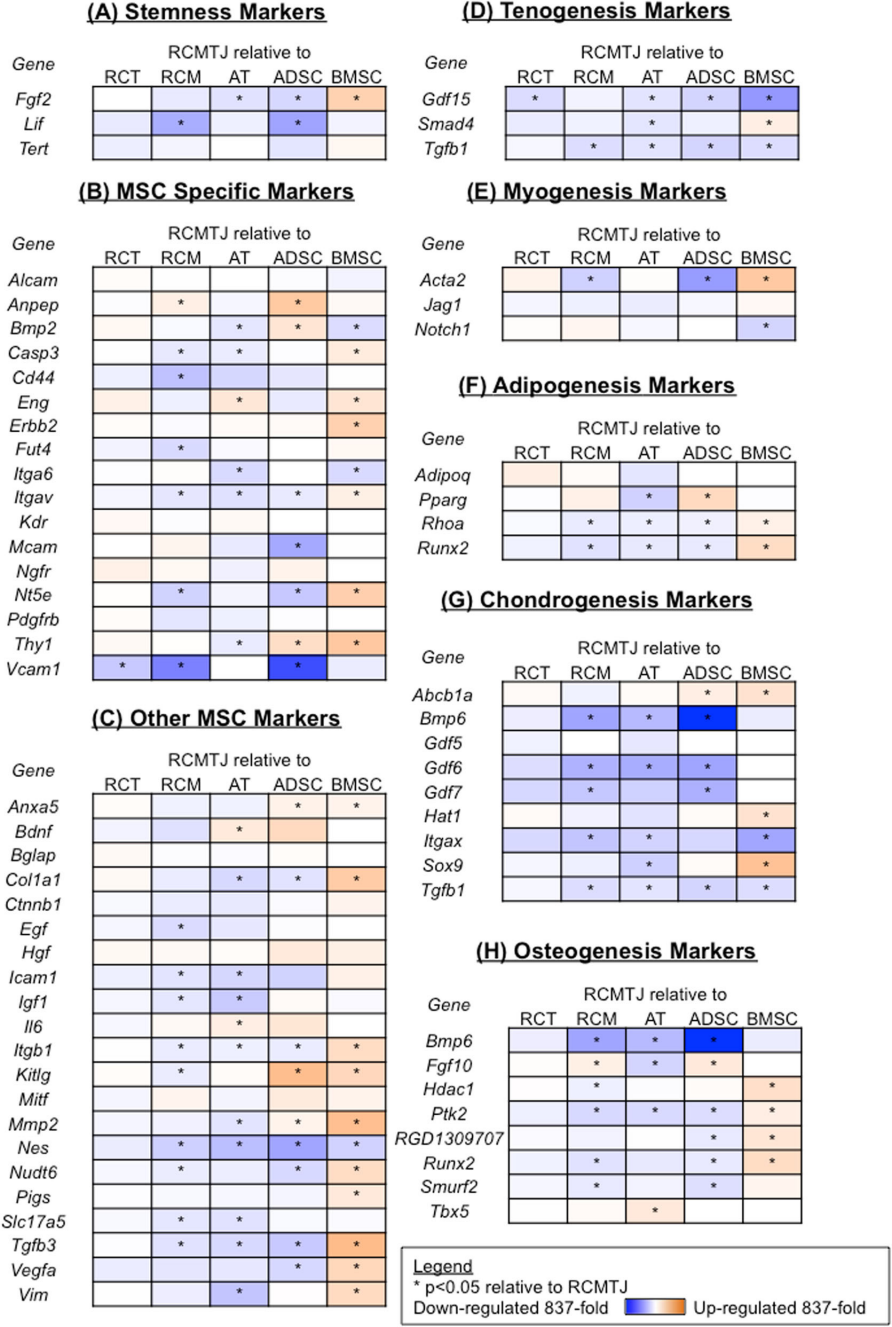
Heat map representation of the fold change in expression of (**a**) stemness markers, **b** MSC specific markers, **c** other MSC markers, **d** tenogenesis markers, **e** myogenesis markers, **f** adipogenesis markers, **g** chondrogenesis markers, and (**h**) osteogenesis markers for the comparison of rotator cuff myotendinous junction cells (RCMTJC) to rotator cuff tendon cells (RCTC), rotator cuff muscle cells (RCMC), Achilles tendon cells (ATC), adipose derived stem cells (ADSCs) and bone marrow derived stem cells (BMSCs). The following genes were not detected: Bmp4, Bmp7, Csf2, Csf3, Fut1, Fzd9, Hnf1a, Ifng, Il10, IL1b, Ins2, Prom1, Ptprc, Pou5s1, Sox2, Tnf and Vwf

We compared the MSC gene expression profile in progenitor cells isolated from six sources [rotator cuff muscle (RCMC), tendon (RCTC) and musculotendinous junction (RCMTJC), adipose derived cells (ADSC), bone marrow derived cells (BMSC), and Achilles derived cells (ATSC; Fig. 5)].

The gene expression of RCMTJCs was very similar to that of RCTCs except for the down regulation of 2 genes, *GDF 15* (marker of tenogenesis) and *Vcam1* (MSC-specific marker). When compared to the MSC gene expression of RCMCs, the RCMTJCs had decreased expression of 26 MSC genes, which included MSC differentiation markers as well as stemness and MSC specific markers. Additionally, one marker of tenogenesis (Tgf-B) and one marker of myogenesis (Acta-2) were down regulated in RCMTJCs but there were no significant differences between the two cell types with respect to other markers of tenogenesis and myogenesis (Smad4, Gdf15, Jag1, Notch1)*.* Two markers (osteogenesis marker *Fgf10,* and MSC specific marker *Anpep*) were upregulated in RCMTJC compared to RCMC.

There were widespread differences in gene expression (up regulation and down regulation) between the RCMTJC and non-rotator cuff derived progenitor cells (ADSC, BMSC, and ATC) for all gene groups including the MSC differentiation markers and MSC specific and stemness markers, which represents the inherent differences due to different tissue origin of these cells.

## Discussion

The results of this study demonstrate that progenitor cells can be harvested from the rotator cuff musculotendinous junction. The MSC gene expression profile of progenitors isolated from the musculotendinous junction was similar to the rotator cuff tendon progenitors but significantly different compared to rotator cuff muscle progenitors (up regulation of 2 genes and down regulation of 26 genes) and also from the progenitor cells isolated from non-rotator cuff tissue (bone marrow, adipose tissue and Achilles tendon).

Although, progenitor cells have been previously isolated from tendon tissue from different anatomic locations including the rotator cuff, this is the first study to demonstrate their presence in the musculotendinous junction [3, 22, 28, 36, 37]. Furthermore, the MSC gene expression profile of the progenitors isolated from MTJ was strikingly different from the MTJ tissue itself, which confirms that these progenitors are a distinct population in the musculotendinous junction of rotator cuff. Zhang and Wang in an in vitro study demonstrated that progenitors isolated from the tendon exhibit distinct properties compared to tenocytes, including differences in cell marker expression, proliferative and differentiation potential, and cell morphology in culture, which is similar to the results found in this study [41].

It has been postulated that engineered tendon tissue has the potential to recapitulate the structural organization of the rotator cuff enthesis but this regeneration will require concerted interplay of multiple players including progenitor cells, growth factors, mechanical fixation, and vascular supply [30, 35, 40]. Progenitor cells have been isolated from healthy tendon (TPC) but factors regulating extracellular matrix production and its regulation are poorly understood [3, 12, 22, 28, 36, 37]. Therefore, the search for ideal progenitor cell for tendon regeneration continues. In this study, we have shown that progenitor cells are present in the rotator cuff MTJ region and are similar to rotator cuff tendon derived progenitors. These findings have future clinical applications in the field of regenerative medicine. In large, massive rotator cuff tears, there is considerable loss of tendon tissue and the residual tendon tissue is degenerative with presence of fatty infiltration, delamination, and poor tissue quality. The MTJ is relatively preserved in the majority of rotator cuff tears and based on the results of our study has potential to be alternate source of autologous progenitor cells for cell based therapeutic strategies for tendon regeneration. Furthermore, the gene expression of MTJ progenitors being similar to tendon progenitors is likely to provide a similar autocrine and paracrine influences on rotator cuff tendon region. However, this hypothesis will require future testing in vivo using animal models and in clinical studies.

Recently, other investigators have explored the gene expression profile of human rotator cuff tendons [5, 9, 17, 22, 32]. Chaudhary et al. demonstrated up regulation of extracellular matrix remodeling genes (some matrix metalloproteinases, and metalloproteinase genes, and disintegrin) and down regulation of some interleukins genes (1, 8 and 27) in tissue samples from human rotator cuff tendon tears compared to normal controls [5]. Choo et al. demonstrated that there was up regulation of genes associated with fatty atrophy and fibrosis in full thickness rotator cuff tears compared to massive rotator cuff tears, which demonstrated down regulation of all gene programs except inhibition of myogenesis [9]. In contrast to aforementioned studies, we have characterized the MSC gene expression profile in progenitors isolated from normal rotator cuff musculotendinous junction without the presence of rotator cuff tear. We found that the gene expression for MSC markers of tenogenesis and myogenesis was preserved among the three cell types isolated from rotator cuff tissue and may reflect the functional similarity between these three cell types (musculoskeletal origin). Interestingly, the expression of adipogenesis markers was significantly down regulated in the RCMTJ progenitors compared to RCM progenitors, ADSCs and BMSCs. The differential in lineage specific gene expressions may simply be a result of the basal level of ‘growth’ factors/cytokines provided by the FBS preparation used, as well as autocrine production of such differentiation factors. Thus, further experimentation in needed to define the similarity/differences in the multipotency as well as the differentiation capabilities of the cells.

The progenitor cells from bone marrow had higher expression of stemness markers and MSC markers compared to MTJ cells. At this stage we have no experiment-based explanation for the differences. However, we hypothesize that each progenitor cell population consists of multiple types, either in terms of lineage or differentiation stage that would include the tissue per se as well as ‘auxillary’ tissue structures such as the peritenon, endomysium, blood vessels etc. A quantitative cell sorting approach followed by gene expression analyses of subpopulations would be needed test this possibility [18].

### Limitations

This study has potential limitations, which should be taken into consideration when interpreting the results. First, the RT-PCR array technology generates vast volumes of data and data interpretation can be subject to interpretive bias depending on the genes tested. However, we have reported our results on all the 84 genes tested to minimize this bias. Secondly, genes can be subjected to post-translational modifications at multiple levels and the up regulation in gene expression may not result in protein translation. Third, the rotator cuff tissue evaluated in this study was from healthy rats without any obvious signs of rotator cuff disease. Therefore, it is possible that gene expression profiles in diseased conditions may be different compared to healthy rotator cuff tissue. Fourth, while the expression profile for stemness and differentiation markers were performed, the differentiation assays to determine how the cells differentiate under certain conditions were not performed. We used the term progenitors for the cultured cells, but they are more of a heterogeneous population of cells. To establish the ‘progenitor cell nature’ of the cultures, they were established following methodologies previously described for progenitor cell culture and expansion from equine, bovine and mouse tissue sources [14, 15]. Finally, the cell populations in rat rotator cuff can be different from human rotator cuff especially given the fact that humans don’t use the upper extremity for ambulatory weight bearing in addition to the inherent biological differences. Furthermore, it is important to note that the MSC markers in rats differ compared to humans.

## Conclusion

This study demonstrates that progenitor cells can be harvested from the musculotendinous junction of a rat rotator cuff. The gene expression of MTJ progenitors is similar to tendon progenitors but considerably different from muscle derived progenitors or other non rotator cuff derived progenitors (adipose, bone marrow and Achilles tendon). However, further detailed experimentation, including cell sorting approaches with stemness markers to obtain populations with differentiation potentials for expression of muscle and tendon specific characteristic is required to further establish clinically relevant use of such cells in the treatment of tendon disorders.

## Data Availability

The datasets used and/or analyzed during the current study are available from the corresponding author on reasonable request.

## Abbreviations

MSCs: Mesenchymal Stromal Progenitor Cells
RC: Rotator cuff
RCT: Rotator cuff tendon
RCM: Rotator cuff muscle
RCMTJ: Rotator cuff musculotendinous junction
AT: Achilles tendon
RT-PCR: Reverse transcriptase-polymerase chain reaction
TPCs: Tendon-derived progenitor cells
DMEM: Dulbecco’s modified eagle medium
FBS: Fetal bovine serum
bFGF: beta fibroblast growth factor
mRNA: Messenger ribonucleic acid
DNA: Deoxyribonucleic acid
Ct: Cycle threshold
ANOVA: Analysis of Variance
ADSC: Adipose derived cells
BMSC: Bone marrow derived cells
ATSC: Achilles tendon derived cells
